# Why Do Some Vertebrates Have Microchromosomes?

**DOI:** 10.3390/cells10092182

**Published:** 2021-08-24

**Authors:** Kornsorn Srikulnath, Syed Farhan Ahmad, Worapong Singchat, Thitipong Panthum

**Affiliations:** 1 Animal Genomics and Bioresource Research Center (AGB Research Center), Faculty of Science, Kasetsart University, 50 Ngamwongwan, Chatuchak, Bangkok 10900, Thailand; farhan.phd.unesp@gmail.com (S.F.A.); worapong.si@ku.th (W.S.); thitipong.pa@ku.th (T.P.); 2 Laboratory of Animal Cytogenetics and Comparative Genomics (ACCG), Department of Genetics, Faculty of Science, Kasetsart University, 50 Ngamwongwan, Chatuchak, Bangkok 10900, Thailand; 3 The International Undergraduate Program in Bioscience and Technology, Faculty of Science, Kasetsart University, 50 Ngamwongwan, Chatuchak, Bangkok 10900, Thailand; 4 Special Research Unit for Wildlife Genomics (SRUWG), Department of Forest Biology, Faculty of Forestry, Kasetsart University, 50 Ngamwongwan, Chatuchak, Bangkok 10900, Thailand; 5 Amphibian Research Center, Hiroshima University, 1-3-1, Kagamiyama, Higashihiroshima 739-8526, Japan

**Keywords:** evolution, karyotype, chromosomal rearrangements, genes, genome

## Abstract

With more than 70,000 living species, vertebrates have a huge impact on the field of biology and research, including karyotype evolution. One prominent aspect of many vertebrate karyotypes is the enigmatic occurrence of tiny and often cytogenetically indistinguishable microchromosomes, which possess distinctive features compared to macrochromosomes. Why certain vertebrate species carry these microchromosomes in some lineages while others do not, and how they evolve remain open questions. New studies have shown that microchromosomes exhibit certain unique characteristics of genome structure and organization, such as high gene densities, low heterochromatin levels, and high rates of recombination. Our review focuses on recent concepts to expand current knowledge on the dynamic nature of karyotype evolution in vertebrates, raising important questions regarding the evolutionary origins and ramifications of microchromosomes. We introduce the basic karyotypic features to clarify the size, shape, and morphology of macro- and microchromosomes and report their distribution across different lineages. Finally, we characterize the mechanisms of different evolutionary forces underlying the origin and evolution of microchromosomes.

## 1. Introduction

The year 2020 was the bicentennial of Charles Darwin’s birth and the 150th anniversary of the publication of his well-known book, “On the Origin of Species by Means of Natural Selection”. One section is entitled, “Organs of extreme perfection and complication” [1], which describes the main features of eye evolution, their prominent position in the body, and their role in developmental and evolutionary biology. Darwin hypothesizes that both a primitive and a complex eye may be evolved from rhodopsin, an ancient molecule, and further explains the effect of eyes on the diversification of life-forms, and the interaction between genetics and developmental biology [1]. Importantly, rhodopsin is found throughout the domain of eukaryotes and is also present in prokaryotes. The rhodopsin family of molecules serves as the photosensitive chemical in all vision systems in creatures across the evolutionary tree, and has been conserved for more than a billion years of life [2,3,4]. The exact nucleotide and amino acid sequences may differ, and the photochemical cascade differs in its details; however, the basic vitamin A aldehyde-protein pairing is still a stable feature. Rhodopsin is also ubiquitous in multicellular animals [5], thus reflecting a basic unification of life-forms in a similar way to genetic material such as chromosomes.

**Figure 1:**
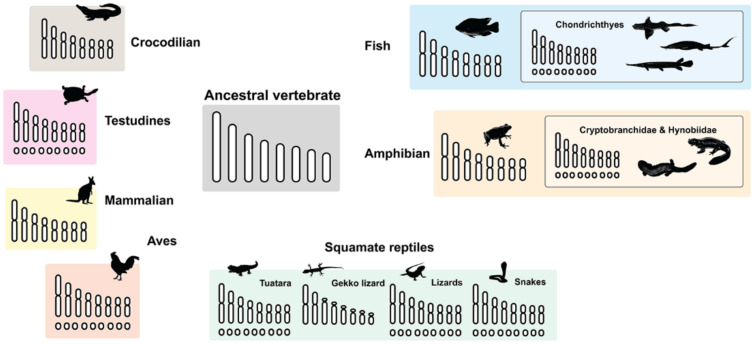
Microchromosome distribution of vertebrates and karyotypic ideograms.

Chromosomes are thread-like structures located inside the nucleus of eukaryotic organisms. Each chromosome comprises DNA moleculaes coiled around proteins, with specific instructions that make each type of living creature unique and is passed equally from parents to offspring. Chromosomes are normally visible under a light microscope only when the cell is undergoing the metaphase of cell division, where all chromosomes are in their highly condensed form, comprising short and long arms and centromeric constriction. Notwithstanding this, in specific lineages of vertebrates, the chromosome set is bimodally characterized by great variations in size and commonly termed as micro- and macrochromosomes, although there is not always a sharp borderline between the two groups (Figure 1). Microchromosomes are a type of smaller chromosome and typical components of avian karyotypes [6]. They have also been observed in some reptilians such as snakes, lizards, and turtles [7,8,9], and in other vertebrates including amphibians [10,11] and fish [12], but they are not found in mammals. Microchromosomes behave like any other chromosomes; they are stably maintained during cell division and have functional centromeres and telomeres [13]. Microchromosomes are also found in insects, particularly in the Belostomatidae family [14,15,16,17,18].

The physical and genetic maps of chickens are the most developed with important international efforts also underway to build a complete genome map similar to what has been done for humans [19,20,21]. Microchromosomes were originally discovered in chicken chromosome preparations, leading to the adoption of the chicken chromosome as the model reference genome, with chromosome size difference and evolutionary linkage homology in vertebrates [22,23,24,25]. Most chicken microchromosomes belong to ancestral linkage groups, resulting in hypothetical ancestral microchromosomes of vertebrates [9,26,27]. The recent emergence of genomics has offered in-depth insights to trace the evolutionary history and unlock the chromosome level mechanisms that might have reshaped ancestral vertebrate evolution [28]. A marine chordate was found to have two successive whole genome duplications (WGD) ~450 million years ago (Mya) before becoming the common ancestor of vertebrates and diversifying into the more than 70,000 species found today [27,29,30]. The separation of *Agnatha* (jawless fish) as the primitive vertebrates and *Gnathostomata* (jawed fish) was found to have four more fusions to form the ancestral Euteleostomi (bony vertebrates) genome of 50 chromosomes [27]. A first version of the ancestral vertebrate pre-2R WGD (2nd round of WGD) protochromosomes was reconstructed using duplicated regions of the human genome, containing four copies of the ancestral diploid vertebrate genome [29,31]. The scenario probably starts from 10–13 protochromosomes, each duplicating twice into 40 chromosomes during the 2R-WGD, but including three repeated fissions and the loss of six chromosomes. The comparison of the lancelet (amphioxus) genome (*Branchiostoma floridae*, Hubbs, 1922) [32] with the genome of several vertebrates defined the unordered gene content of 17 ancestral chordate linkage groups of the last common chordate ancestor, which revealed a global four-fold conserved linkage homology with vertebrate genomes [33,34]. Surprisingly, when gar and chicken genomes were compared, almost half of the gar karyotype (14/29 chromosomes) showed a one to one relationship with chicken chromosomes, including microchromosomes [27,35]. Thus, microchromosomes may be ancestral features in Euteleostomes, which in turn raises a scenario for their origin through 2R duplications. However, in this setting, chromosome evolution would tend to favor fusions into fewer and larger chromosomes, as found in mammals [36]. Therefore, “How can we predict evolutionary direction and the crucial impact of microchromosomes?” Returning to the rhodopsin story, Darwin states, “It would be best to trace the gradual development of complex eyes in the ancestors of presently known animals. Since the fossil record does not allow this, we should look to the entire spectrum of eyes in various extant species. We can emphasize that these changes, although happening gradually through the immensity of geological time, could occur by the power of natural selection” [37,38]. However, evolution could change one part of an organism without interfering with the integrity and survivability of the organism as a whole. A similar idea is now applied by current evolutionary biologists for microchromosomal implications and origins. 

In light of this whole scenario, the critically intriguing question is why such a high proportion of small chromosomes have persisted in specific lineages. Is it possible that the existence of many small chromosomes represents a specialized system for information storing? Taking advantage of data sourced from the recently achieved milestone of comparative genomics of several vertebrate genomes, we review the evidence pertaining to the origin and different genetic profiles between macro- and microchromosomes in vertebrates. Hopefully, this evidence will help answer the main research question of why some vertebrates have macro- and microchromosomes.

## 2. How Can We Clarify Karyotypic Features of Micro- and Macrochromosomes?

It remains difficult to make definite counts of the number and morphology of microchromosomes based on reports of different vertebrate karyotypes, because no standard rules exist for the identification of macro- and microchromosomes [6,39,40]. Generally, microchromosomes, as a particular type of very small chromosomes, are morphologically indistinguishable as dots on metaphase plate chromosomes [9,39,41,42]. All microchromosomes arrange with each homologous pair, and several reports have attempted to measure the actual size of microchromosomes by scale bar or computationally as digital pixels [43,44,45]. However, the size of each microchromosome varies depending on the condensation state of each metaphase cell, creating difficulty to precisely organize the pattern [46,47]. The classical criterion to define microchromosomes is their size, which varies between research groups from 0.5 to 1.5 µm [28,40,45,48,49]. When crudely determining the category of chromosomes, the centromeric position of microchromosomes cannot be defined by conventional staining such as orcein or Giemsa, whereas macrochromosomes can be designated as metacentric, sub-metacentric, sub-telocentric, and acrocentric. The chicken genome contains 2*n* = 78 chromosomes ranging in size between 250 Mb (the largest macrochromosome) [50] and 3.5 Mb (the smallest microchromosome) [6,51], and a total genome size of 1.1 Gb [20,52]. Chicken chromosomes are classified arbitrarily into two major groups: the macrochromosomes ranging from 30 to 250 Mb (chromosomes 1–8 and the sex chromosomes (ZZ, male or ZW, female)), whereas the remaining smaller chromosomes are microchromosomes of, on average, 12 Mb in length (the smallest being 3.5 Mb) [20,51]. In addition to chicken microchromosomes, other amniote groups possess microchromosomes generally <30 Mb in length [6,25,53,54]. In reptiles, microchromosomes in snakes (such as *Naja naja*) can attain a small size of around 10 Mb; in turtles (such as *Chelonia mydas*), the smallest microchromosomes recorded were 7.8 Mb in length; whereas in lizards (such as *Lacerta agilis*) the minimum microchromosomes are 12 Mb in size (https://www.ncbi.nlm.nih.gov/assembly/?term=Reptiles, accessed on 1 July 2021). Genome sequencing of many vertebrate species now provides unprecedented detail sufficient to compare microchromosomes across diverse lineages.

## 3. Microchromosome Distribution in Vertebrate Lineage 

Large microchromosome distribution is observed across the vertebrate clade, with variable microchromosome numbers ranging from one pair of microchromosomes in various lizard species such as Bosk’s Fringe-fingered lizard (*Acanthodactylus* spp.), lacertid (*Lacerta* spp.), racerunner lizard (*Eremias* spp.), and snake-eyed lizard (*Ophisops elegans*) to more than 40 pairs in Arctic lamprey (*Lethenteron camtschaticum*) [28,55,56,57,58] (Figure 1). The occurrence of a diversity in chromosome number between different vertebrates presents an opportunity to correlate chromosome evolution with the timing and types of events [27].

Bird karyotypes are generally composed of ~80 chromosomes. Of these, 7 or 8 pairs of the largest chromosomes are macrochromosomes ranging from 3–6 µm in length, whereas the remaining 30 to 32 pairs are microchromosomes of 0.5–2.5 µm in length [39,42,59,60]. Apart from the Falconiformes (falcons) and the Psittaciformes (parrots), which have reduced diploid numbers with fewer microchromosomes [61], there is neither a gradual reduction nor an increase of microchromosome numbers or total length during long term evolution in birds [6]. 

The bimodal karyotypic feature is also observed in turtles that have chromosome numbers of 2*n* = 28–66 [62,63,64], with a range of macrochromosomes between 10 and 36, and up to 56 microchromosomes [58] (Table 1). Despite such variability, karyotypes are presented for 13 of the 14 genera of side-necked turtles (suborder Pleurodira, families *Pelomedusidae* and *Chelidae*). Pelomedusids have low diploid numbers and few microchromosomes (2*n* = 26–36); the five largest chromosomes are homologous in the three genera. Despite this substantial homology, some pericentromeric regions of macrochromosomes can also show interspecific chromosomal differences. For example, a comparative analysis of satellite sequences among the four sea turtle species including *Chelonia mydas*, *Caretta caretta*, *Eretmochelys imbricata,* and *Lepidochelys olivacea* showed species-specific variation of microsatellites in heterochromatin regions [65]. Chelids have a high diploid number and many microchromosomes (2*n* = 50–64) and are similar in this respect to cryptodires (2*n* = 50–66). The sea turtle species (*Cheloniidae*) showed a diploid number of 56 chromosomes, arranged in 11 bi-armed chromosome pairs (1–10 and 12) and 17 acrocentric pairs (11 and 13–28; 13–28 were microchromosomes), and FN = 78.

In Lepidosauromorpha (tuataras and squamate reptiles), tuataras have a diploid chromosome number of 2*n* = 36, consisting of 14 pairs of macrochromosomes and 4 pairs of microchromosomes [85,86], whereas squamate reptiles show substantial karyotypic variability with a diploid number of chromosomes ranging from 2*n* = 20–68 [87]. This high variation is arguably driven by dynamic repeated fusion of macro- and microchromosomes. Furthermore, certain squamate reptiles may harbor very few or no dot-shaped microchromosomes, for example, in lacertid lizards (Lacertidae) and geckos (Gekkonidae) [56,58,88,89,90,91,92,93,94,95,96,97,98,99,100], whereas other squamates can carry as many as 36 microchromosomes [99]. The most common chromosome number in snakes is 2*n* = 36, comprising 16 macrochromosomes and 20 microchromosomes, while worm lizards show a large variation in chromosome number (2*n* = 30–50) [88,100]. The extant lizards (*Lacertilia*) also exhibit a large variation in both chromosome number (2*n* = 24–46) and chromosome morphology [88] (Figure 1). By contrast, the crocodile karyotype contains chromosomes with the absence of microchromosomes [9,101,102]. Considering avian and non-avian reptiles, the question arises as to why geckos and crocodiles do not contain a microchromosomal structure, despite being in the same evolutionary line. 

Apart from amniotes, most fish have between 40 and 60 chromosomes, while some (holocephalian, chondrostean, holostean) showed karyotypes with microchromosomes [53,103]. In most fish families (*Anostomidae*, *Prochilodontidae*, *Curimatidae*), microchromosomes are often present as accessory elements [104], such as in *Astyanax mexicanus* whereby microchromosomes occur as supernumerary B chromosomes [105,106]. Furthermore, it is possible that microchromosomes are present in the karyotypes of many primitive vertebrates [10,11,53,69]. Lastly, the chromosomal frequency of freshwater fish is higher than marine fish, suggesting that karyotypic dynamics can change with species habitat [103].

Interspecies changes in karyomorphic and chromosomal frequency, with different numbers of microchromosomes, have also been observed in amphibians such as primitive species of Urodela, Anura, and Apoda [70,72]. Nevertheless, the rate of chromosomal rearrangements in amphibians was previously estimated to be less frequent compared to mammals [107]. Recent genomic studies have shown that chromosomes experienced high levels of fusion type rearrangements in salamanders and frog species [108,109]. Further studies are required to investigate whether this high-level tendency of chromosomal rearrangements in amphibian genomes can trigger the formation of microchromosomes, and how such forces might impact their evolution. Although the distributions of microchromosomes in some vertebrate groups are more well-studied, the advent of molecular cytogenetic, genomic, and bioinformatic approaches has offered the opportunity to test long-standing hypotheses in both model and non-model taxa.

## 4. Differences in Characteristics of Macro- and Microchromosomes

**Figure 2:**
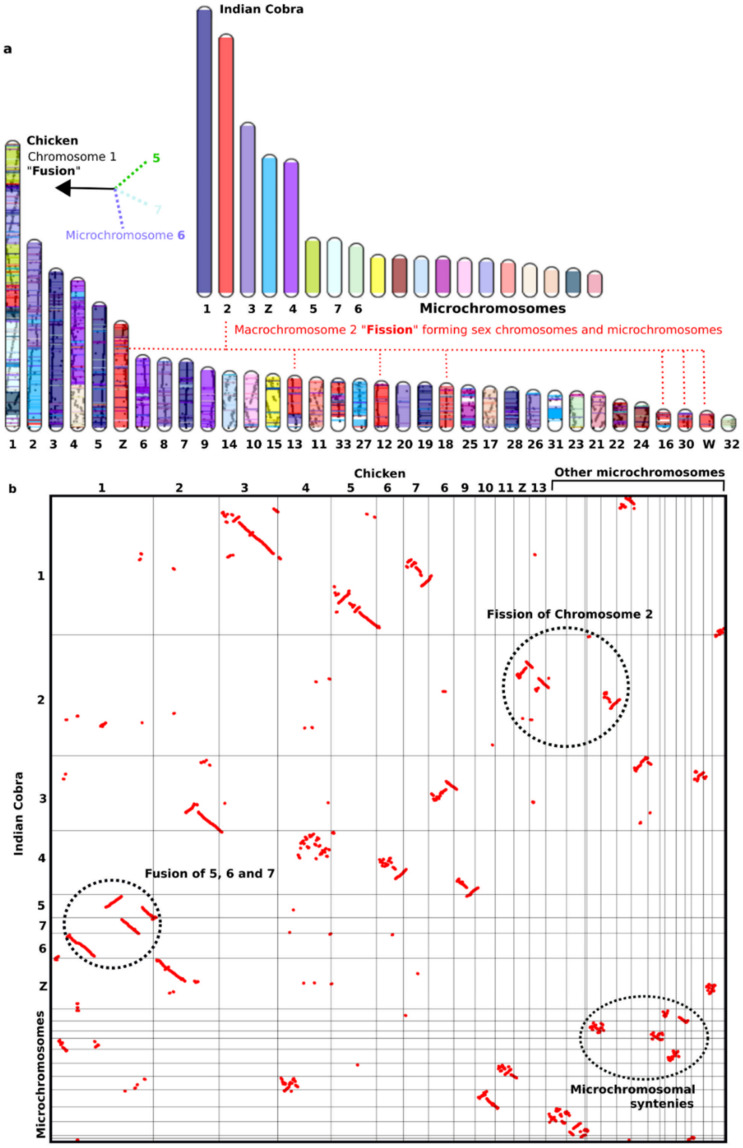
Cross-species homology relationship of microchromosome syntenies and inter/intra-chromosomal rearrangements for the analyzed species. (**a**) In silico chromosome map of Indian cobra and chicken chromosomes. Same colors correspond to syntenic regions between different chromosomes. (**b**) A dot-plot view of genomic comparisons indicate different evolutionary patterns of chromosomal rearrangements, such as fusion, fission and microchromosomal homologies.

Vertebrate microchromosomes consistently exhibit many distinct features across lineages, including high gene density and high rates of recombination, thus representing a functionally and evolutionarily unique fraction of the genomes [20,52,54,110,111,112]. Chicken microchromosomes are GC-rich, contain CpG islands, comprise 50% of all genes derived from the level of methylation, and are 2–3 times as gene-dense as the macrochromosomes [22,39,52,113,114,115,116,117,118]. By contrast, the macrochromosomes are AT-rich and exhibit weak R-, C-, and T-banding [39,119]. Generally, the GC-content of chromosomes increases exponentially with the reduction in chromosomal size (ICGSC, 2004 [52,120,121]; however, a few exceptions are evident. For instance, this tendency is not seen in most teleosts [122], whereas primitive fish such as lamprey show a significant association between GC% and chromosome sizes. Furthermore, amphibians, e.g., salamander and frog species (*Ambystoma mexicanum*, *Xenopus laevis*, *X. tropicalis*), do not show a correlation between GC% and chromosome size either [122]. This indicates that GC% increases together with genome size in these instances, suggesting that lineages comprising several microchromosomes might be the counterparts of mammalian GC-rich chromosomal segments. Researchers [115] attempted to address this statistically by analyzing chicken chromosomes that were outliers to the “smaller chromosome size = more gene-dense” rule. Chromosome size-dependent GC heterogeneity seems to be a widespread characteristic in sauropsids (avian and non-avian reptiles), whose karyotypes consist of microchromosomes, and possibly originated from the common ancestor of sauropsids [40,74,123,124]. Previous studies demonstrated by comparative genomics that chromosomes have been highly conserved between the chicken and the turtle [76,125,126]. For instance, the karyotype of the Chinese soft-shelled turtle (*Pelodiscus sinensis*, Wiegmann, 1835) [127], which consists of 9 pairs of macrochromosomes and 24 pairs of microchromosomes (2*n* = 66), is very similar to the chicken karyotype [128]. Furthermore, it is assumed that around 50% of total gene contents are localized on the microchromosomes in avian genomes [6,43,117,129]. Chicken microchromosomes are also considered to extensively retain the ancestral linkage groups of genes [117]. Moreover, GC-poor genes are two to three times more likely to reside on macro- than on microchromosomes in both chicken and turtle genomes, whereas GC-rich genes tend to reside equally on macro- and microchromosomes [40]. Concurrently, several types of microchromosome-specific repeated sequences are reported [128,130,131] in turtles and avians. Since these microchromosome-specific repeated sequences are GC-rich, it is possible that heterochromatic regions also contribute to the high GC-content in microchromosomes as well as regions where functional genes are coded. Moreover, a chromosome size-dependent GC heterogeneity was also identified in the red-eared slider turtle (*Trachemys scripta elegans,* Wied-Neuwied, 1839) [132] and the Nile crocodile (*Crocodylus niloticus*, Laurenti, 1768) [133] using a chromosome flow sorting technique [123]. The GC portion that encompasses genomic regions (over the scale of several kb to Mb) forms the "isochore" which has been extensively reported in crocodiles and turtles [134,135,136]. Recently, these isochores have also been identified in teleost genomes with similar characteristics. Researchers [135,137,138] analyzed the GC-content of exonic third codon positions (GC3) of more than 6000 expressed sequence tags (ESTs) in the American alligator (*Alligator mississippiensis*, Daudin, 1802) [139] and mentioned that the alligator genome has a certain level of GC heterogeneity suggesting the presence of GC-rich isochore in ancestors of archosaurs (birds and crocodilians). The GC content of alligator and crocodile assembled genomes was examined, and a higher average GC content was observed compared to many other vertebrates [140]. In this analysis, substantial standard deviation in GC content across assembled scaffolds suggested the presence of GC-rich isochores, indicating the heterogeneneity of the alligator genome. Snake karyotypes have also been highly conserved within this group. The usual diploid number is 2*n* = 36, consisting of 8 pairs of macrochromosomes and 10 pairs of microchromosomes [95,96,97,98]. The effect of large differences of karyotypes, especially the number of microchromosomes between the snake and the other two species, might be considered. The chromosome number is largely different from the chicken karyotype because of the remarkable difference in the number of microchromosomes. This suggests that chromosomal rearrangements have occurred more frequently in the snake lineage than in chicken-turtle lineages, resulting from frequent repeated fusions between macro- and microchromosomes and between microchromosomes [82,94] (Figure 2). In total, 11 chromosomal segments homologous to chicken microchromosomes were localized to snake macrochromosomes [123]. However, snake microchromosomes contain a higher proportion of GC-rich genes than macrochromosomes, as observed in both the Chinese soft-shelled turtle and chicken [54,123,141]. This suggests that macrochromosomes tend to contain more GC-poor genes, whereas microchromosomes tend to contain more GC-rich genes. The correlation coefficient of GC is also lower between the rat snake (*Elaphe quadrivirgata*, Boie, 1826) [142] and chicken orthologs than between the Chinese soft-shelled turtle and chicken [123]. This might occur because the phylogenetic distance is larger between the snake and chicken than between the turtle and chicken (Figure 1).

Chromosomal reshuffling can trigger changes in chromosome sizes and differences of GC levels, thus, causing exchange of genes between macro- and microchromosomes. This phenomenon was revealed from substantial homology between chicken microchromosomes and snakes macrochromosomes, which harbor several orthologs in both lineages. The first reptilian species for which the whole genome sequence was released was the green anole lizard (*Anolis carolinensis*, Voigt, 1832 [143]) [144], although with recent trends in genomics, 16 reptilian genomes have now been assembled at the chromosome level and annotated at NCBI (https://www.ncbi.nlm.nih.gov/assembly/?term=reptiles, accessed on 10 May 2021). Genomic trends for other vertebrate groups are also increasing, with a total of 187 and 13 accomplished chromosome level assemblies for species of fish and amphibian, respectively (https://www.ncbi.nlm.nih.gov/assembly/?term=reptiles, accessed on 10 May 2021). The green anole lizard, whose karyotype consists of 6 pairs of macrochromosomes and 12 pairs of microchromosomes [144,145] does not show such marked biases in GC-content between macro- and microchromosomes. Anolis has a homogeneous genome composition compared with other amniotes [144,146] and, unlike the chicken, the GC-content is similar between macro- and microchromosomes. These results make it possible to infer global GC heterogeneity of the leipidosaurian genome and the shift of GC-content caused by chromosomal rearrangements during the sauropsid evolution. Lepidosauria is a species-rich group consisting of over 10, 000 extant species, and the karyotypes are also diversified within the group [56,82,84,89,90,92,93,95,96,97,98,147]. Chromosome size-dependent GC heterogeneity has probably disappeared in the specific lineage [123]. By contrast, there is no significant correlation between the GC content and the size of chromosomes that harbor them in the human and the mouse [148], consistent with analysis at the genomic level. It is still not clear whether this is a function of their small physical size or greater gene density in microchromosomes (thus, greater ability to access transcriptional machinery) [149]. Accordingly, chromosomal size-dependent GC compartmentalization seems to be unique to sauropsids, with most karyotypes consisting of macro- and microchromosomes. By contrast, chromosome sizes are relatively uniform and there is no striking bias in inter-chromosomal GC-content in most mammals. These facts indicate that sauropsids adopted chromosomal size-dependent GC compartmentalization strata, whereas mammals maintained the system in which GC-rich and -poor regions coexist on individual chromosomes in a highly juxtaposed manner. Although the intra-genomic comparison of GC content across mammals, birds, and non-avian sauropsids (i.e., reptiles) revealed a similar pattern of GC heterogeneity [146], it remains unclear whether this GC heterogeneity was derived from a common ancestor or the result of a convergence that occurred independently across these lineages. This hypothesis has yet to be verified by further large-scale studies, not only in turtles, but also in other sauropsids. Several mammalian species such as shrews, microbats, tenrecs, and rabbits have experienced an increase in GC content, as marked by the identification of the most GC-poor and -rich classes of genes [148]. It is important to clarify whether monotremes, marsupials, and amphibians have a similar pattern of intra-genome GC distribution to eutherians (to speculate on the ancestral configuration for the amniote genome) by adding outgroup polarity to the present scheme. A comparison of conserved non-coding sequences (CNSs) among different vertebrates revealed that mammals tend to have highly GC-enriched flanking regions around these CNSs [84]. Further research should clarify whether the GC-enriched regions on microchromosomes are adaptive or merely the consequence of neutral evolutionary processes. Further insight into the evolution of genome structural features (such as GC regions) may spur novel studies assessing the evolutionary benefit of gene contents localized on microchromosomes.

## 5. Independent Recombination Frequency between Macro- and Microchromosomes as a Driver to Change Chromosome Structure

Recombination facilitates the successful inheritance of chromosomes during meiosis, which plays an important role in reshaping the evolutionary dynamics of organisms [150]. Comparison of the physical size of chromosomes and the rate of recombination (crossing over) reveals a strong negative relationship in birds and mammals [111]. Recombination density is higher in smaller chromosomes such as microchromosomes, resulting in an increased mean recombination rate in birds and non-avian reptiles [20,39,151]. Recombination rates are higher on microchromosomes (median rate, 6.4 cM/Mb) than on both intermediate (3.9 cM/Mb) and macrochromosomes (2.8 cM/Mb) [20,110]. The rate of recombination varies considerably between different genomic regions and is most evident between macro- and microchromosomes. The higher recombination rate on small chromosomes ensures that pairing of chromosomes occurs during meiosis [39]. This might result from the basic requirement of at least one chiasma per chromosome per meiosis, possibly facilitated by a higher density of cohesin binding sites. Cohesin can bind densely in centromeric regions of chromosomes, where it helps mount sister chromatids onto spindle microtubules from opposing poles (biorientation), thus facilitating recombination [152]. It is believed that high densities of cohesin binding sites increase the chance of formation of the synaptonemal complex in these regions, and result in a higher rate of recombination [152,153,154]. Cohesin holds the sister chromatids together during the metaphase and ensures their successful segregation during cell division [155]. Moreover, meiotic recombination may tend to initiate in the accessible chromatin at gene promoters [156]. This initiation of each meiotic recombination activity occurs with the programmed formation of a DNA double-strand break (DSB), which can be repaired either as a “crossover” or as a “non-crossover” [157,158]. The domestic chicken (*G. gallus*) has been well studied for its highest recombination rate among birds. High frequency of recombination might be due to artificial selection during domestication, as also observed in the greylag goose (*Anser anser*, Linnaeus, 1758 [159]) [111,160,161]. Although earlier studies suggested an increased recombination rate in domesticates, a comprehensive later study on mammals confirmed that artificial selection does not drive the evolution of an increased recombination rate in domestic mammals. By contrast, individual bivalents of macrochromosomes had approximately the same average number of MLH1 genes (MutL homolog 1) as in the white wagtail (*Motacilla alba*, Linnaeus, 1758 [162]). The total numbers of crossovers in SC1-8 are 34.2 and 34.5 in the wild wagtail and the chicken [163], respectively. However, the wild wagtail microchromosomes had about one-quarter more crossovers than their chicken homologs (42.2 and 31.2, respectively). This indicates that exceptionally high recombination rates exist in the wild avian taxa. In reptiles, the comparison of the exact level of recombination at taxonomic scale is lacking, but the overall tendency of chromosomal rearrangements might suggest a high frequency of recombination [84]. It remains unexplored to which extent individual avian microchromosomes can vary in recombination rate across this lineage. Therefore, further availability of chromosome-level avian genome assemblies could unlock this information.

Similarly, recombination is closely linked to gene conversion, which has been shown to be biased toward elevating the GC content [164]. Increased GC content might have a positive effect on the expression of genes within that region, favoring the accumulation of highly expressed housekeeping genes over the larger more complex genes involved in development and transcriptional regulation [165,166]. In mammalian chromosomes, gene density differences are correlated with chromosome banding patterns as the R-bands (gene-rich) have higher gene densities than the G-bands (gene-poor) [156]. The R- and G-band regions of chromosomes are also interlinked with different aspects of nuclear organization and gene regulation [167]. Increased acetylation of the amino terminus of histone H4 is observed in transcriptionally active regions [168,169,170]. The distribution of acetylated H4 in human and hamster chromosomes has been shown to be non-random, with hyperacetylation of R-bands [171] and hypoacetylation of heterochromatic domains [172]. The avian microchromosomes also share many characteristics with these mammalian R-bands, such as high gene density, high CpG island content, and early replication in the S phase [113,116]). Microchromosomes are hyperacetylated and most replicate early in the S phase, typical characteristics of gene-rich chromosomal domains [114,116]. To find an indication of genes-rich domains on microchromosomes, histone acetylation studies provide a method for visualizing regions of high gene content of the genome that is independent of sequence characteristics [173,174]. Furthermore, preferential staining of microchromosomes with antibodies against acetylated H4 also provides strong evidence for elevated gene density. As previously observed, microchromosomal H4 is acetylated at multiple lysine residues [116]. This correlation between higher recombination rate/gene conversion and higher gene density suggests an evolutionary pressure for an increase in gene density on microchromosomes.

Studies in mammals report a positive correlation between GC content with both the substitution rate [175,176,177,178] and levels of genetic variation [179]. It is likely that the increased prevalence of hypermutable CpG dinucleotides in GC-rich sequences is an important factor in increasing mutation rates in these regions [180]. A further potential correlate of substitution rate is the local recombination rate [181,182,183,184], which can be highly variable even at small scales on human chromosomes [185]. This could be due to a direct causal effect, resulting from the erroneous repair of double-strand breaks that initiate recombination [186]. However, local recombination rates also correlate with GC content [187], an observation argued to result from recombination driving the evolution of GC content [188]. Notably, many genomic features that differ between macro- and microchromosomes have been implicated to cause mutation rate variation [165,182,189,190,191,192,193,194,195,196]. When comparing microchromosomes with macrochromosomes, the results indicated 18% higher average sequence divergence in introns and 26% higher average rate of synonymous substitutions in coding sequences. In general, selective constraint is expected to homogenize differences caused by mutation rate variation. Although the presence of extended splice-sites and potential regulatory elements means that intron sequences could be subject to evolutionary constraint, selective constraint is unlikely to result in differences between intronic rates on macro- and microchromosomes. Moreover, as introns on microchromosomes tend to be shorter [20,52], a greater proportion of sequence is likely to be comprised of regulatory elements, which would result in a reduction in substitution rates on microchromosomes. 

Mutations in CpG dinucleotides are an important factor for explaining the high divergence of microchromosomal intron sequences. Methylated CpG sites easily deaminate, resulting in C→T transitions possibly 10 times more frequent than other mutations [197]. A number of further effects could result in differences in substitution rates between avian autosomes, including recombination being mutagenic. Base composition may directly alter regional substitution rates if global rates of AT→GC and GC→AT mutations differ, although the effect is largely dependent on the GC content equilibrium that a sequence is evolving toward [198]. Reptilian genomes such as turtles were reported to have lower substitution mutation rates compared to mammals and birds [199,200,201]. This also provides them with a gene-dense structure consisting of microchromosomes with three to four times shorter intergenic sequences than on macrochromosomes. Furthermore, intergenic distances as well as the average size of the introns on microchromosomes are lower, resulting in a much higher gene density compared with macrochromosomes [8,25]. Indeed, these findings resurrect the question of whether the rate of recombination is generally higher in birds compared to other vertebrates [20,52], due to a higher proportion of microchromosomes and the relatively small size of avian genomes. A recent population genomics survey has identified a strong heterogeneity in recombination rates along the green anole genome [202]. Moreover, the latest findings provided evidence that macrochromosomes of vertebrates including snakes feature a high recombination rate [112]. Examining the recombination landscape in rattlesnakes using population genomic data identified rapidly evolving hotspots with activity of PRDM9 that can direct meiotic recombination. A general caveat in studies of recombination and genomic parameters is that while estimates of recombination rates reflect the contemporary situation, most genomic parameters (substitution rates, GC composition, and microchromosomal organization) are the result of long-term evolutionary processes.

## 6. Nuclear Organization of Macro- and Microchromosomes

Based on the visual inspection of fibroblast and neuronal nuclei of chickens, macrochromosome territories were located mostly toward the nuclear periphery, while microchromosome territories formed a few distinct clusters located toward the nuclear center, leading to the radial arrangements of macro- and microchromosomes [43]. In chickens, microchromosomes appear to cluster in a central position in the interphase nucleus, with the macrochromosomes occupying the nuclear periphery [43,124,203]. This localization correlates with the state of recombination frequency on chromosomes. Such reports have shown that most vertebrates exhibit a reduced recombination rate in chromosome centers relative to chromosome peripheries of nuclear architecture [204,205]. Low recombination frequency was observed in the telomeric regions of examined animals including ray-finned fish (*Actinopterygii*), birds, insects, and mammals [205]. The crossover rate was significantly lower in the center of chromosomes relative to their telomeric peripheries. The preferential position of mid-late replicating chromatin is at the nuclear periphery and the central position of early replicating chromatin, also previously observed in mammalian cell nuclei [206,207]. The reduction of recombination rates in macrochromosome centers of the zebra finch (*Taeniopygia guttata*, Vieillot, 1817) [208] is more extreme than in other birds [111,209], while the white wagtail macrochromosomes exhibited a clear U-shaped distribution of recombination frequencies, adding another example of comparatively reduced recombination in the centers of nuclear architecture [210]. By contrast, neighborhoods between non-homologous as well as homologous macrochromosome territories (side-by-side arrangements) are variable [43]. Epigenetic mechanisms, including DNA-methylation and histone acetylation, play an apparent role in higher-order chromatin architecture and gene expression [211,212]; however, their potential contribution to the intranuclear arrangements of chromosome territories has not been studied. In contrast to early replicating gene-dense chromatin, gene-poor mid-to-late replicating chromatin may carry binding sites for the reconstituting nuclear lamina during telophase [213]. This could push early replicating gene-dense chromatin into a more interior position, also observed in mammalian cell nuclei [207,214]. Furthermore, late-replicating chromatin has been observed around the nucleoli. Microchromosomes are predominantly early replicating with a small proportion of late-replicating segments [116,119,215]. A specific radial chromatin arrangement exists with preferential positioning of gene-dense early replicating chromatin in the nuclear interior and gene-poor late replicating chromatin at the nuclear periphery, which seems to be an evolutionarily conserved motif for the organization of the nucleus in both chicken and human cells.

Nevertheless, the stable genome organization of macro- and microchromosomes is highly conserved, with each ancestral microchromosome preferentially locating in the center at interphase [40,43]. Remarkably, these microchromosomes still maintain their central position in the nucleus even when recently fused to a larger chromosome (as in falcons and parrots) [43,124,216]. Furthermore, chromosomal arrangements noted in mitotic cells correlate to some extent with chromosome territory arrangements in interphase nuclei. However, microchromosomes may lack the necessary motifs to bind lamin proteins no matter what the karyotypic configuration [28]. It is possible, therefore, that these motifs subsequently accumulate on fused microchromosomes. Nonetheless, we would expect pressures against this: the internal gene-dense microchromosomes could provide access to transcription factories and safely keep genes away from the silencing environment of peripheral heterochromatin [217,218]. It is also possible, although unlikely, that the macrochromosomes lose their lamin attachments. Modeling chromatin dynamics suggests that the entire nuclear organization can invert when this tethering is interrupted [219]. Some chromatin must remain tethered to the nuclear periphery, implying that the macrochromosomal sequence will also be conserved. Recent comparative genomic analysis of Hi-C sequencing data from multiple vertebrate lineages has shown that microchromosomes can exhibit significant levels of interchromosomal interactions and seem to be colocalized within the central nuclear territory [40]. Similar patterns of high level interchromosomal interactions for microchromosomes were also observed in chicken [220] and rattlesnake [54] genomes, and our expanded sampling indicates that these patterns are likely remarkably consistent across diverse vertebrate lineages. This analysis further suggests that microchromosomes might harbor a higher proportion of open chromatin than macrochromosomes. This model of nuclear organization represents a genomic configuration that has existed since early vertebrate evolution. Cytological observations have shown that microchromosomes in all lineages are spatially separated into a central compartment at interphase and during mitosis and meiosis. This reflects higher interaction between microchromosomes than macrochromosomes, as observed by chromosome conformation capture, and suggests some functional coherence. In highly rearranged genomes, fused microchromosomes retain the most ancestral characteristics but these may erode over evolutionary time. Surprisingly, de novo microchromosomes have rapidly adopted high interaction.

## 7. Distribution of Repeated Sequences between Macro- and Microchromosomes

Repeated sequences are a major source of homologous sites in chromosomal rearrangements between and within chromosomes [81,221,222,223,224,225]. Repeated sequences are mainly classified into tandem repeats such as satellite DNA (satDNA), mini-satDNA, and micro-satDNA [225,226,227], with interspersed repeats as transposable elements (TEs) [228]. A popular formation of repeated sequences is telomeric repeats, in which microchromosomes might have gained telomeric repeats preferentially as observed from the high intensity of telomeric sites on microchromosomes in birds [229]. Similar cases are also observed in several squamate reptiles [91]. This occurrence of telomeric repeats is generally rare in the macrochromosomes of turtles, but FISH mapping showed brighter signals on microchromosomes, indicating high abundance [230]. The rate of recombination might be associated with repeats such as telomeric repeats. Some regions may be functions of the initial copy number and the rate of recombination [231]. Molecular cytogenetic studies on meiotic chromosomes of the Armenian hamster suggested that interstitial (TTAGGG)_n_ signals coincided with chiasmata, the sites of meiotic exchange [232]. The ability of telomeric sequences to promote recombination was also shown in yeast [233]. The enrichment of avian microchromosomes with (TTAGGG)_n_ repeats provides additional evidence for telomere-associated recombination. In birds, microchromosomes always show a higher rate of recombination than macrochromosomes [6,111,151]. This might imply that microchromosomes of squamate reptiles also present with a higher frequency of recombination than macrochromosomes. It is tempting to consider the unusually frequent occurrence of (TTAGGG)_n_ sequences as an important element in explaining the high recombination rate in species comprising microchromosomes such as birds and squamate reptiles. Unexpectedly, in the W sex chromosome of the lacertid lizard, (TTAGGG)_n_ sequences are abundant on microchromosomes comprising the entire chromosome [234]. This might relate to the process of sex chromosome differentiation [235,236,237]. Both the almost complete coverage of some microchromosomes with telomeric repeats and the presence of large telomeric arrays at one chromosomal end in another subset of microchromosomes may be caused by the amplification of (TTAGGG)_n_ repeats on these tiny chromosomes. 

SatDNA repeats are fast-evolving sequences which can constitute highly repeated and/or highly conserved monomers in eukaryotic genomes ranging from 150–400 bp in length [225,238]. However, satellite diversity and abundance are difficult to identify because of repeat complex structures [239]. Due to reduced genome size, avian genomes are characterized by considerably lower percentages of repeats compared to other vertebrates [240]. In different species of birds such as *Colaptes melanochloros* (Gmelin, 1788 [241]) (2*n* = 84) and *Colaptes campestris* (Vieillot, 1818 [242]), SatDNA repeats are accumulated with centromeric and telomeric regions in both macro- and microchromosomes along with clusters of 18S rDNA [243]. In the Chinese soft-shelled turtle (*Pelodiscus sinensis*, Wiegmann, 1835 [127], family *Trionychidae*), a novel satellite designated PSI-Bgl was cytogenetically characterized and mapped on microchromosomes in both the centromere regions and satellite arms, but not detected on macrochromosomes [244]. This site-specific satellite compartmentalization pattern is also observed in the Mexican musk turtle (*Staurotypus triporcatus*, Wiegmann, 1828 [245]) and the giant musk turtle (*S. salvinii*, Gray, 1864 [246]) [247]. By contrast, satellites have been studied in snakes [248], lacertids [55,249,250,251,252,253,254] scincids [255,256], and varanids [257,258]. All satellites studied were localized to chromosomal heterochromatin, while predominantly in centromeric, pericentromeric, and/or telomeric regions. In Lacertinae, different types of satDNA repeats are characterized into different satellite families including species and genus-specific sub-families [259]. Among these, the centromeric HindIII family containing two subfamilies (I and II) constitutes 5–10% of the genome. Another family known as TaqI, possesses only interstitial sites with 2.5–5% of the genome. Differences in abundance, chromosomal position, and evolutionary rate were observed for the HindIII and TaqI families across lacertids. One novel AAN-TaqI satellite with an AT-enriched monomer of 187–199 bp was isolated from populations of the Atlas dwarf lizard (*Atlantolacerta andreanskyi*, Werner, 1929 [260]) [254]. In varanids, the VSAREP satellite has been identified in the water monitor (*Varanus salvator macromaculatus*, Deraniyagala, 1944 [261]) and is conserved in the genomes of Asian and Australian varanids, but not in African varanids [258,262]. This satellite family is considered to play an important role in chromosomal rearrangement in varanid lineages [258]. Three different types of heterochromatic region-linked satellite families are found in the Burmese python and habu snakes [234]. These satellite families include: (1) PFL-MspI (168 bp) from *Protobothrops flavoviridis* (Hallowell, 1861 [263]), (2) PBI-DdeI (196 bp), and (3) PBI-MspI (174 bp) from *Python bivittatus* (Kuhl, 1820 [264]). Thongchum et al. (2019) studied 40 snake species to gain an improved understanding of the conservation of PBI-DdeI satellite evolution and function. Results suggest that size-specific compartmentalization might have occurred in turtles and birds, but not in squamate reptiles [248]. Lineages with no microchromosomes like crocodiles show satDNA distribution in the centromeric region of all chromosomes. The satellite families CSI-HindIII and CSI-DraI isolated from the Siamese crocodile (*Crocodylus siamensis*, Schneider, 1801 [265]) were characterized in the crocodile genome, indicating their localization in the heterochromatic blocks of centromeres [102]. The CSI-HindIII family is conserved across all extant crocodile lineages of *Crocodylidae*, whereas the CSI-DraI satellite is known only in *Crocodylus* and is not represented in other crocodile genomes. A genome with a low degree of compartmentalization, which would show limited recombination and a low frequency of chromosomal rearrangements, appears to have been preserved in squamate reptiles. This observation is based on the size-specific amplified compartmentalization of satellites, such as microchromosome-specific satellites in turtles yet not in squamate reptiles. Accumulation and conservation of repeats resulted in an increase in chromosome size and number of non-deleterious insertion sites—two features that would have further hampered recombination and chromosomal rearrangements [62]. It would be interesting to determine the crucial impact of chromosomal compartmentalization with species diversity for geckos, lacertids, and the remaining groups of squamate reptiles with both macro- and microchromosomes. Crocodylia, which shows low species richness, rarely exhibits genome rearrangements among members. This suggests that the ancestral crocodilian karyotype was highly conserved with no microchromosomes [82,102]. The rate of chromosomal rearrangements may reduce over evolutionary time until genomic stability and optimal karyotypes are achieved. It is hypothesized that both compositional and structural factors of repeats may drive reptilian karyotypic evolution, with a transition from the heterozygous to the homozygous phase through a series of rearrangements. For an improved understanding of the underlying mechanisms, characterization of the specific types of rearrangements, such as cryptic inter- or intrachromosomal changes, and comparative genomic analyses in conjunction with cytogenomics or chromosomics are required to investigate genome structure across diverse reptile lineages [42]. To increase our knowledge of the dynamics and comparative landmarks of repeats, further in-depth studies are required to, firstly, understand how the scale of variability of these elements drives genome evolution, and secondly, how such variation affects processes such as gene regulation, sex chromosome evolution, and karyotype reorganization between macro- and microchromosome lineages.

Furthermore, examination of additional reptilian species is needed to elucidate the mechanisms of microchromosome inheritance during evolution. Most repeat sequences are derived from TEs, and at least 50% of the vertebrate genome appears to be derived from these sequences; researchers [193,266] compared the genome sizes of birds and found a narrow range of DNA content (2–4 pg), smaller than that found in any other vertebrate class. The questions arise: Is this due to a monophyletic origin of birds from a small ancestral genome? Or is it DNA loss due to an evolutionary constraint on genome size in birds? The monophyletic origin of birds from a small ancestral genome is supported by early studies on primitive fish [53], which suggested an ancestral genome of 50% of a typical mammal and a karyotype with microchromosomes. If this is true, there would be no need to propose a drastic loss of DNA during the evolution of avian microchromosomes. Why then did the avian genome remain so small and not increase in size like the genomes of mammals and reptiles? Differences in the DNA content of vertebrates are mainly due to differences in repeat content [267]. Amphibians have characteristically huge genomes, often 10 times larger than a typical mammalian genome, and repeat contents of 50–90%. In contrast, mammals and reptiles are not so extreme and have repeat contents of 30–50%, whereas birds have the lowest with only 15–20% repeats. These observations would suggest that the genome sizes of mammals, reptiles, and birds have been more constrained than amphibians. Furthermore, the avian genome appears to be the most constrained. Most repeat sequences are derived from TEs [222,267]. Recently, the ancestry and approximate age of TEs in the human genome were inferred from a phylogenetic analysis of the genome sequence [193]. This analysis showed that the mammalian genome increased in size 50–150 Mya through an accumulation of transposons, comprising at least 50% of the human genome today. This analysis also showed that transposon activity virtually stopped during the past 35–50 million years. In birds, the CR1 repeats are the major retrotransposon family. Sequence analysis reveals that the avian CR1 repeat family is ancient and dying out, with about 50,000 poorly related sequences in avian genomes [268]. This is consistent with a minor role for TEs in the evolution of genome size in birds. It would be interesting to determine repeatomic variation ranges in microchromosomes of vertebrates groups, and the extent of variation at species and genus levels. Significant advances are possible through large-scale species sequencing and genome assembly.

## 8. Highly Conserved Linkage Homology between Macro- and Microchromosomes and the Fusion-Fission Model of Vertebrate Evolution

The genomes of all presently existing vertebrate species have diverged from a common ancestor over a period of several hundred million years. Comparative genomics between remote species is a key tool for the delineation of evolutionary ancestral syntenies and the process of chromosomal rearrangements [56,82,89,90,91,92,269]. To find evidence that some or all of the chicken microchromosomes arose by a process of chromosome fission or fusion, an outgroup species is needed to define the ancestral linkage homologies [259]. All the evidence suggests that some microchromosomes were already present in the common ancestor that gave rise to birds and other terrestrial vertebrates [6]. Presumably, a process of chromosome fission created the remaining avian microchromosomes [270]. Cross-species analysis on over 70 avian species from 15 different orders has revealed a remarkable lack of inter-macrochromosomal rearrangements. Microchromosomes are present in all avian lineages and are assumed to be of adaptive value; otherwise, some researchers propose that they would have been lost by chance during the 100–250 million years of avian evolution [271]. The fission model predicts that avian microchromosomes may represent ancestral linkage homologies, provided that there have not been any chromosome rearrangements in this lineage [23,272,273,274]. Certain birds, such as the Coraciiformes, only have a few microchromosomes. Furthermore, in Falconiformes, New World falcons have 42–45 pairs of microchromosomes, whereas Old World falcons only have 12–13 pairs [275]. Chromosomal rearrangements such as fusions and fissions would have disrupted these ancestral linkage homologies. Similar cases are also observed from non-avian reptiles. Molecular phylogenetic analyses have suggested that extant sauropsids (reptiles and birds) are divided into two major groups, the lineages of Testudines (turtles), Archosauria (crocodilians and birds), and Lepidosauria (tuatara, lizards, worm lizards, snakes), although the phylogenetic position of Testudines is still debatable [275,276,277]. Most sauropsidan species have karyotypes consisting of macro- and microchromosomes similar to birds, except for crocodilian species whose karyotypes contain no microchromosomes [8,9,28]. Microchromosomes were first recorded in iguanid and teiid lizards [58,278] and are considered to have originated from fragments of ancestral macrochromosomes [279]. Different reptiles possess varying numbers of microchromosomes in their chromosomal sets, and these karyotypic differences are important in reptile comparative analyses for investigating their genetic makeup [280]. As stated earlier, comparative genomic analyses reveal that genetic linkages were highly conserved between avians and reptilians [26,56,82,89,90,91,92,95,96,97,98,144,281]. Several crocodile and gecko chromosome pairs are composed of chromosomal segments homologous to turtle and the majority of squamate reptile microchromosomes [56,82,89,90,91,92,95,96,97,98,144,235]. By contrast, the macro- and microchromosomes of turtles are counterparts of those found in chickens, suggesting that the ancestral karyotype of Archosauromorpha, probably composed of at least eight pairs of macrochromosomes and many indistinguishable microchromosomes, has been highly conserved for more than 250 million years following their divergence from Lepidosauromorpha [94,123,125]. Chicken and red-eared slider (*Trachemys scripta elegans*, 2*n* = 50) [282] macrochromosomes are remarkably well conserved, considering that these species shared a common ancestor (the Archosauromorpha ancestor) over 200 Mya [283]. Interestingly, the karyotypic features of the Gila monster, *Heloderma suspectum*, were described by Pokorná et al. (2014) consisting of 2*n* = 36 chromosomes (14 macro- and 22 microchromosomes), similar to Iguania and snake karyotypes (http://chromorep.univpm.it, accessed on 1 July 2021) [284,285]. A series of chromosomal fusion-fission events (centric fusion-fission, tandem fusions, insertion, and transposition), followed by centromere inactivation events between macro- or other microchromosomes, resulted in diversified karyotypes among squamate reptiles [84,89,90,235]. The phylogenetic placement of reptiles and birds in the presence or absence of microchromosomes suggests that the ancestral karyotype of reptiles might have contained both macro- and microchromosomes [26,144]. The microchromosomes might have disappeared by fusion between macro- and microchromosomes and/or between microchromosomes in the lineage of crocodiles. Copious evidence from BAC mapping confirms cross-species chromosomal homologies reflecting the occurrence of ancient chromosomes in an ancestral genome at least 400 Mya [95,97,98]. These would be cases of linkage homology disruption in the avian/reptilian lineage, either through the process of chromosomal fission or fusion. Previous research has analyzed “former” microchromosomes (i.e., those that have since fused in evolution to become part of a larger chromosome) such as those seen in Falconiformes [286,287], which largely retain their inherent microchromosomal properties such as gene density, GC content, and recombination rate in larger chromosomes [20]. In lizards (anoles) and snakes such as the Indian cobra, microchromosomal fusion has also been observed (Figure 2). Similarly, whole-genome comparisons between chickens and snakes reveal a high level of chromosomal syntenies and rearrangements. For example, the macrochromosome 1 of the Indian cobra (*N. naja*) has substantial homology with two macrochromosomes and two microchromosomes of chickens, indicating ancestral macrochromosomal fission (Figure 2).

In addition to amniotes, other vertebrate genomes including some primitive amphibians and lower bony fish also represent a highly dynamic number of microchromosomes. The karyotypes of *Cryptobranchidae* and *Hynobiidae* families of amphibians can carry 2*n* chromosomes ranging from 56–66, with 14–19 pairs of microchromosomes [11,69] Chromosomal linkage homologies, as well as fission and fusion rearrangements have been detected between avian and amphibian genomes, and comparative mapping showed a considerable amount of homology between different macro- and microchromosomes [109,288]. Microchromosomes can also be found in chondrostean and holostean fish (2*n* = 46–112), related to crossopterygian fish that gave rise to terrestrial vertebrates 280 Mya, with genomes similar in size to birds [53]. Lower chordates such as sea lamprey can carry exceptionally high numbers of microchromosomes, with diploid karyotypes consisting of 168 small dot-like chromosomes [289,290,291]. The genome-wide comparison of sea lamprey has identified ancestral conserved orthologous groups with two chicken macrochromosomes. Further analysis is required to investigate the homology of microchromosomes at different taxonomic levels of vertebrates [292]. The ubiquitous distribution of microchromosomes across different vertebrate lineages suggests that microchromosomal rearrangements appear to be characteristic of the common ancestor of terrestrial vertebrates. Furthermore, the marine chordate genome experienced 2R WGDs ~450 Mya before becoming the common ancestor of vertebrates and diversifying into the more than 60,000 species found today [27]. After the separation of *Agnatha* (jawless fish), the most primitive of vertebrates and *Gnathostomata*, four more fusions took place to form the ancestral *Euteleostomi* genome of 50 chromosomes. However, when the 2R-WGD occurred in chordates and how many rounds of WGDs occurred after 2R is still being debated. One study suggested that 2R-WGD might have occurred at the base of vertebrates and a 3R-WGD was followed in lampreys [293]. However, the most recent analysis based on genome comparisons of the lamprey, chicken, and gar genomes provided evidence of only a 2R-WGD at the base of the vertebrates, followed by chromosome-scale duplications in lampreys [292]. One intriguing concern regarding the 2R-WGD was raised by comparing microchromosomes of gar and chicken [35]. In total, 12 gar and chicken microchromosomes shared considerable homology and can parsimoniously be considered ancestral to *Euteleostomi*. Their distribution in the tetrads resulting from the 2R does not follow a noticeable pattern, i.e., they are distributed among all tetrads more or less randomly. Therefore, from this comparison, it is likely that microchromosomes did not originate from a set of pre-2R microchromosomes, but only started evolving after the 1st WGD. Other studies have suggested that they emerged as an ancestral karyotype ~400 Mya in the ancestral vertebrate karyotype [6]. Bioinformatic reconstructions of avian microchromosomes have shown that they correspond directly with gnathostome ancestor protochromosomes [29], suggesting that they have remained remarkably unchanged throughout evolution. Comparative gene mapping between the genomes of chicken, human, mouse, and zebrafish revealed evidence that microchromosomes might be 400 million years old. A recent study has proposed that the typical avian-like karyotypic pattern of microchromosomes mostly emerged before birds and turtles diverged and was present in the theropod dinosaur lineage [294]. Nonetheless, the exact estimate of microchromosome origin remains unclear and further investigation of chromosome-scale assemblies using modern omics tools will be crucial to obtain in-depth insights. Unraveling the enigma of vertebrate evolution will require a deeper understanding of temporal changes in linkage homology that affect chromosome structure and function, as well as how these changes influenced and are influenced by gene and genome evolution. Knowledge gained from studying chromosome homologies will further facilitate comprehension of mechanisms that describe and drive evolutionary trajectories in vertebrates.

## 9. Natural Selection and Adaptive Value for the Existence of Microchromosomes in Specific Lineages

The gross structure and organization (at karyotypic level and in interphase nuclei) of the genome of any species have broad functional significance. The number and shape of chromosomes as well as the order of genes thereon can impact evolution in relation to phenotype and variation of that species. Amniotes diverged in a relatively short period 250–300 Mya [295,296]. When considering the karyotypic variation of vertebrates, there may be an evolutionary advantage in maintaining a karyotypic structure comprised many compact and gene-rich [294,297]. Such microchromosomes were present in all avian lineages 100–250 Mya and are assumed to be of adaptive value [271]. The interchromosomal fusion-fission processes appear to be the main driver, creating small metacentric chromosomes [76,298,299]. Such variation in chromosome number suggests that microchromosomes are not of any adaptive value, because most avians have 30–35 pairs of microchromosomes; hence, the process that created them must have acted before bird radiation 100 Mya [300] as an evolutionary advantage by retaining this signature avian configuration. The high frequency of microchromosomes in testudines, which represent the primitive lineage of Archosauromorpha, suggests that birds and turtles have retained the ancestral state of Archosauromorph karyotypes under similar patterns of evolutionary pressure [94]. Microchromosomes in snakes carry relatively less abundance of repeats than macrochromosomes, and analysis of localized genes enriched on microchromosomes in rattlesnakes (such as venom genes) have shown that the selection of multiple gene families through multiple tandem duplication events might have driven the evolution of microchromosomes [54]. Turtles, as ancient reptiles (older than snakes and crocodiles), have a large number of microchromosomes, suggesting that birds retained the ancestral state of Archosauromorph karyotypes, whereas snakes have relatively lower number of microchromosomes compared to turtles and birds. By contrast, microchromosomes are lacking in the crocodilian lineage and geckos. This evolutionary dynamics of microchromosomes, with varying occurrence across different vertebrate taxa, indicates that natural selection might have fixed these elements in each specific lineage. Several hypotheses might be postulated as to why only specific lineages contain microchromosomes.

Firstly, the primitive vertebrates that gave rise to the avian lineage had a genome size and a repeat content similar to advanced birds, and a karyotype with about 20 pairs of microchromosomes. Generally, these chromosomal changes can be rapid as proposed by the model of stasipatric speciation [24,301,302]. The rate of chromosomal rearrangement depends on both the rate of chromosomal mutation and the rate of fixation, while the rate of mutation depends on the frequency of homologous segmental sites [24]. The rate of fixation depends on many evolutionary forces, including selection, inbreeding, and genetic drift [24,221,301,303]. Fixation of chromosomal rearrangements during the evolutionary process created and shaped macro- and microchromosomes in specific lineages, but not randomly. If the distribution of chromosome fissions in the population were random, then the distribution of chromosome sizes in birds, turtles, and snake would tend toward one with a few large chromosomes, macrochromosomes, and many smaller chromosomes and microchromosomes. The presence of microchromosomes would suggest that a few intra- and interchromosomal rearrangements reached fixation. Notwithstanding this, cell size and genome size are correlated in vertebrates [304]. The ancestral genomes of birds, snakes, and lizards remained small or may have been reduced further in size [266,297]. Avian cells are generally smaller than mammals, and smaller cells have a higher rate of oxidative metabolism. This theory is also supported by a significant correlation between genome size and the ability to fly in mammals and birds [134]). Avian genomes may be constrained by the energetic needs for flight as a possible adaptive response. Similarly, extreme morphological and physiological adaptations in reptiles such as snakes and turtles seem to be driven by genome-wide structural variations and gross chromosomal rearrangements [84,287,294]. These evolutionary mechanisms reshaped the evolution of genes under positive, negative, and neutral selection. In the Burmese python, a high number of genes, functionally related to developmental processes, have been detected that experienced positive selection in ancestral snakes [305]. It remains unknown whether the majority of these positively selected genes were localized on microchromosomes that served as a genomic reservoir to facilitate the evolution of adaptive traits. Evidence of positive selection had recently been obtained by genome sequencing of the Komodo dragon (*Varanus komodoensis*, Ouwens, 1912 [306]), and positively selected genes have been identified in pathways related to energy metabolism, cardiovascular homoeostasis, and hemostasis [307]. Birds, turtles, and some squamate reptiles showed a high number of microchromosomes in their respective genome sizes of ~1.2–1.6, 1.4–2.2, and 1.8–2.2 Gb (Table 1). The net effect of these constraints has been minimization of the repeat content in birds and turtles, while the need for a higher recombination rate on microchromosomes is another constraint that has resulted in the divergence of the properties (GC-content, repeat-content, and gene-density) of macro- and microchromosomes, thus resulting in a reduction in the DNA content of microchromosomes. With repeat-poor genomes, birds and turtles have lower potential for intrachromosomal rearrangement, and fusion/fission events are most likely. The karyotype of the common ancestor of extant sauropsids is thought to have contained both macro- and microchromosomes [6,85] although some lineages underwent frequent secondary fusion of microchromosomes resulting in no or few microchromosomes as seen in geckos, crocodiles, and the avian order Falconiformes [308,309]. If these rearrangements are fixed, they will be expected to scramble the gene content of their chromosomes and equalize the size of chromosomes. This may have been the case for most mammals, amphibians, and some reptiles with the complete absence of microchromosomes. Mammals (as descendants of reptiles), geckos, as well as crocodiles and amphibians with their larger and repeat-rich genomes have the potential to undergo more intra- and interchromosomal rearrangements. Ancestral genomes that gave rise to amphibian, reptilian, and mammalian lineages increased in size due to the spread and amplification of TEs. This is supported by the size of extant genomes, the fossil record, and the sequence of repeat-rich genomes such as human [193,266,267,310]. Essentially, this is an extension of the chromosome-based model of chromosomal rearrangement [311] in which the products of chromosome fission remain as independent chromosomes. Therefore, avian microchromosomes may be a by-product of an evolutionary process that minimizes the repeat content and genome size of birds, rather than for any specific adaptive value of these chromosomes.

Secondly, as the comparative genomic data shows, macro- and microchromosomes are derived from the same set of ancestral chromosomes given that microchromosomes code for 50% of all chicken genes which were reported sharing orthologs with various genomic regions of human [20]. The recombination frequency on microchromosomes is higher than that found in mammalian chromosomes. Furthermore, a high rate of recombination is thought to be necessary to ensure correct pairing of microchromosomes during mitosis and meiosis [110]. The need for a higher recombination rate would also have been a strong selective pressure leading to divergence in the properties of macro- and microchromosomes. Recombination rates vary broadly across populations, species, and higher taxonomic levels, suggesting that they may contribute globally to patterns of biological diversification [312]. Recombination characteristics can directly influence the processes of population divergence and speciation [313,314,315,316]. Therefore, we might expect variation in recombination rates to contribute to distinct speciation patterns observed across taxa. For instance, extensive morphological diversification sometimes coexists with shallow genetic divergence between populations. Several examples are known in birds, where striking plumage differences are characterized by little or no differentiation in molecular markers throughout most of their genomes (e.g., *Vermivora* warblers) [317,318,319], and *Lonchura* munias [320]. In other organisms such as snakes, genomic regions can undergo lineage-specific relaxation of selective pressure on certain genes, for instance, the Hox and Tbx limb-patterning genes support fossil evidence for successive loss of forelimbs and then hindlimbs during snake adaptive evolution [321]). Selection promoting or maintaining divergence at a few key genomic regions and gene flow homogenizing selectively neutral variation are considered the major drivers of such patterns [319]. Nevertheless, crossover frequency and distribution determine which traces, selection, and gene flow are left in genomic landscapes [322]. It is therefore plausible that a pattern of marked phenotypic diversification coexisting with a lack of genome-wide divergence can be associated with high recombination rates. However, empirical support for this connection has yet to be found. The white wagtail (*Motacilla alba*, Linnaeus, 1758 [162]) is a widespread passerine bird. The population structure and differentiation in molecular markers in this species are broadly incongruent with geographical variation in plumage signals, a pattern that was appropriately named “messy speciation” in a recent review of literature pertaining to speciation genomics [323]. Reasons for its long-lived success are in the realms of speculation but might be due to its ability, facilitated by many chromosomes including microchromosomes with high recombination rates, to generate variation which is thought to be the driver of natural selection. This means that a larger number of small chromosomes inherently generate variation through increased genetic recombination in addition to random chromosome segregation. Variation in meiotic recombination, such as differences in the frequency and genomic distribution of crossover events, has fundamental effects on evolutionary processes [312]). These characteristics shape associations between alleles at independent loci, thereby influencing the rate of evolutionary responses, the fate of new beneficial mutations, and the effectiveness of selection against deleterious mutations [314,324,325,326,327,328]. Despite the fact that a single crossover on a microchromosome would shuffle fewer functional genetic elements compared to a crossover on a macrochromosome, the effective gene density might be approximately doubled in the microchromosomes of birds [117]. Variation, in turn, facilitates adaptation and may have contributed to the wide phenotypic variation seen in birds, turtles, and snakes. By contrast, the tendency toward reduction of the number of microchromosomes in certain species might reflect an increase in genetic variation caused by microchromosomal fusion. However, the type of genetic material in the microchromosomes is still misunderstood and it remains to be proven that fusion of microchromosomes and more intra-microchromosomal crossover increase genetic variation as a selective advantage. However, it is plausible that microchromosomes might somehow drive species richness. For instance, living crocodilians lacking microchromosomes include only 27 species, while extant crocodilian diversity is low [329]. Furthermore, the mechanisms involved in the changes of the GC-content of the genes after the fusion of microchromosomes into the macrochromosomal complement remain unknown. It has been suggested that the GC-content is primarily influenced by local recombination rates via GC-biased gene conversion [188,312]. Under this model, A or T is displaced by G or C through mismatch repair when an AT/GC heteroduplex is formed at recombining regions. Accordingly, AT/GC heterozygotes produce more GC than AT gametes, thus conferring predominance of GC alleles in frequently recombining regions. Recombination rate is negatively correlated with the size of chromosome arms in the human and chicken genome [160,330]. Unfortunately, it is difficult to distinguish between causative and secondary effects shaping the pattern of recombination, but it remains likely that some or all of these features work synergistically.

Thirdly, intervening sequences are on average 3–4 times shorter in GC-rich than in GC-poor isochores in the chicken genome [331]. If causally related, then selection for a high recombination rate in microchromosomes will continue to increase their GC-content, reduce gene size, and repeat content, and increase gene density. A similar hypothesis has been proposed by [331] to explain why intervening sequences (and therefore genes) are smaller in GC-rich isochores. Many papers reported existing correlations between gene function and base compositions of the genes, the genomes, and the promoter regions [332,333,334,335,336,337]. The difference in global GC-content between macro- and microchromosomes may potentially cause a biased distribution of gene functions between the chromosomes: some proteins containing more amino acids for GC-rich codons due to functional constraints may be more advantageous in being encoded in micro- than in macrochromosomes. By analysis of dN/dS ratios, we demonstrated that the proteins of genes located on microchromosomes are more evolutionarily conserved. This echoes findings from a mouse-rat comparison [177] in which the dN/dS ratio was found to be negatively correlated with GC content (and hence, recombination rate). Two potential hypotheses have been invoked to explain this observation: it could represent local variation in the efficacy of natural selection, which is known to covary with recombination rate [327,338] or it could indicate that microchromosomes are enriched for slowly evolving genes that fulfill conserved functions, such as housekeeping genes.

## 10. Conclusions

Vertebrate karyotypic evolution has been extensively investigated by molecular cytogenetic techniques, providing interesting insights to unearth information about the dynamics of macro- and microchromosomes. This review emphasized the unique characteristics of microchromosomes, discussing important evolutionary aspects about their genomic origin, composition, and organization. These features suggest that microchromosomes represent ideal karyotypic components for housing genes underlying vertebrate evolution and adaptation. With the rapid development in genome sequencing technologies and advancements in bioinformatics tools, now is the ideal time to integrate cytogenetics and genomic approaches to decipher the mechanisms responsible for reshaping vertebrate genomes. Huge impacts are already being made through the availability of chromosome-level assemblies for diverse vertebrates. These resources will provide opportunities to test hypotheses related to the role of microchromosomes in the genome evolution, the relevance of their genetic prevalence, and the mechanisms that drove the evolutionary shift from solely macrochromosomal systems to those carrying both types of chromosomes.

## Figures and Tables

**Table 1:** Range of genomic features in different classes of vertebrates.

Vertebrate Groups	Diploid Chromosome Range	Macrochromosome Range	Microchromosome Range	Genome Size Range (Gb)	References
Testudines	26–68	10–36	0–56	2.7–5.4	Valenzuela and Adams [63]
Crocodilian	30–42	-	-	1.3–3.9	Srikulnath et al. [9]
Fish	12–168	12–60	0–144	0.3–17.05	Gregory [66]; Gregory and Witt [67]; Arai [68]
Holocephalian, chondrostean and holostean fishes	58–112	24–64	34–52	2.98–14.8	Ohno et al. [53]
Amphibian	18–106	18–92	2–30	0.93–137	Morescalchi [69,70]; Voss et al. [71]; Schmid et al. [72]; Perkins et al. [73]
Cryptobranchidae and Hynobiidae	40–78	20–50	30–40	16.5–56.8	Morescalchi [10,11]; Zhang et al. [74]
Aves	40–142	20–60	10–90	0.96–2.2	Organ et al. [75]; Tegelström et al. [42,76]; Kapusta et al. [77]
Mammalian	6–102	6–102		1.6–6.3	Ferguson-Smith and Trifonov [78]; Graphodatsky et al. [79]; Kapusta et al. [77]
Squamate Reptiles	20–68	10–42	0–56	1.1–5.4	
Tuatara	36	28	8	4.9	Gregory et al. [80,81]; Srikulnath et al. [82]; Deakin and Ezaz [58]; Pasquesi et al. [83]; Ahmad et al. [84]
Gecko lizard	26–68	10–36	-		
Lizards	20–62	10–38	0–28	1.03–3.8	
Snakes	26–50	10–38	0–36	1.3–3.7	
